# Safety and feasibility of 3D‐electroanatomical mapping‐guided zero or near‐zero fluoroscopy catheter ablation for pediatric arrhythmias: Meta‐analysis

**DOI:** 10.1002/joa3.13062

**Published:** 2024-05-16

**Authors:** Gusti Ngurah Prana Jagannatha, I. Made Putra Swi Antara, Anastasya Maria Kosasih, Jonathan Adrian, Brian Mendel, Nikita Pratama Toding Labi, Wingga Chrisna Aji, Bryan Gervais de Liyis, Made Refika Widya Apsari Tangkas, Yosep Made Pius Cardia, Alif Hakim Alamsyah

**Affiliations:** ^1^ Faculty of Medicine Udayana University/Prof. Dr. I.G.N.G Ngoerah General Hospital Denpasar Bali Indonesia; ^2^ Division of Electrophysiology and Cardiac Pacing, Department of Cardiology and Vascular Medicine Udayana University/Prof. Dr. I.G.N.G Ngoerah General Hospital Denpasar Bali Indonesia; ^3^ Department of Cardiology and Vascular Medicine, Sultan Sulaiman Government Hospital Serdang Bedagai North Sumatera Indonesia; ^4^ Faculty of Medicine Sam Ratulangi University Manado North Sulawesi Indonesia; ^5^ Faculty of Medicine Muhammadiyah Yogyakarta University Yogyakarta Indonesia

**Keywords:** Ablation, pediatric, near‐zero fluoroscopy, zero fluoroscopy

## Abstract

**Background:**

Catheter ablation in the pediatric population using fluoroscopy has been known to cause adverse events. This study aims to assess the effectiveness and safety of zero fluoroscopy (ZF) and near‐ZF‐guided catheter ablation for the treatment of arrhythmias in the pediatric population.

**Methods:**

The PubMed, Embase, and Cochrane library databases were searched and reviewed for relevant studies. Outcomes of interest include safety, short‐term, and long‐term effectiveness. We classified patients ≤21 years old who underwent ZF or near‐ZF ablation with fluoroscopy time ≤1.5 min as our study group and patients within the same age range who underwent conventional fluoroscopy and/or near‐ZF ablation with a mean fluoroscopy time >1.5 min as our control group. Both ZF and near‐ZF ablation utilized 3D‐electroanatomical mapping (3D‐EAM).

**Results:**

Ten studies composed of 2279 patients were included in this study. Total fluoroscopy time (MD –15.93 min, 95% CI (−22.57 – (−9.29), *p* < .001; *I* ^2^ = 84%)) and total procedural time (MD –22.06 min, 95% CI (−44.39 – (−0.28), *p* < .001; *I* ^2^ = 88%)) were significantly lower in the near‐ZF group. Both ZF and near‐ZF demonstrated a trend towards improved success rates compared to conventional fluoroscopy but did not achieve statistical significance for all subgroup analyses. Ablation in the study group also decreased incidence of complication compared to the control (RR 0.35; 95% CI (0.14–0.90); *p* = .03; *I* ^2^ = 0%).

**Conclusion:**

ZF and near‐ZF ablation reduced the overall duration, compares in effectiveness, and shows a superior safety profile compared to control group.

## Graphical abstract

The summary of success rate, complication rate, and long‐term efficacy of 3D‐electroanatomical mapping‐guided zero or near‐zero fluoroscopy catheter ablation.

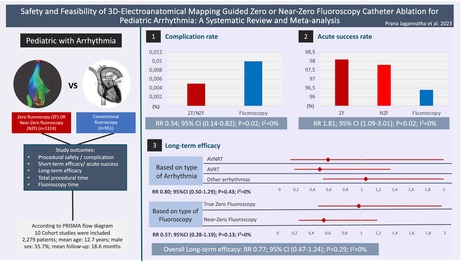

## 1 INTRODUCTION

Catheter ablation, established as the primary treatment modality for a majority of tachyarrhythmias, is associated with inherent risks, prominently manifest through the use of fluoroscopy.^1^ This effect is pronounced in the pediatric population, who are up to 10 times more likely to develop complications, leading to increased long‐term morbidity and reduced quality of life.^2^ The American College of Cardiology Task Force recommends the acronym ALARA, which stands for “as low as reasonably achievable”, as a guiding principle for cardiovascular imaging, thus limiting the dose of exposure to ionizing radiation.^3^ Pediatric cardiac anatomy complicates ablation, primarily related to vascular access challenges and the risk of inadvertent damage to vital structures in the relatively small heart.^4^, ^5^, ^6^ Unique pediatric arrhythmias and faster conduction further demand procedural modifications.^7^, ^8^

Recent technological advances have led to the development of 3D‐electroanatomical mapping (3D‐EAM) systems that have made it possible to significantly reduce fluoroscopy or eliminate it altogether, thereby reducing radiation exposure during ablation.^9^, ^10^, ^11^, ^12^ According to a recent trial, zero fluoroscopy (ZF) in supraventricular ablation shows a 96% risk reduction in cancer‐related events and deaths.^13^ Nevertheless, further research is required to determine the safety, effectiveness, and recurrence rate of arrhythmias caused by 3D‐EAM‐guided zero or near‐zero fluoroscopy catheter ablation in the pediatric population. More research is required to optimize the procedure in this group, including understanding the differences in outcomes for distinct types of arrhythmias using ZF and near‐ZF ablation compared to conventional procedures. This systematic review and meta‐analysis aim to prove the effectiveness and safety of ZF and near‐ZF‐guided catheter ablation in the pediatric population.

## 2 METHODS

This systematic review followed the Preferred Reporting Items for Systematic Reviews and Meta‐analyses (PRISMA) guidelines.^14^ The protocol is registered in the international prospective register of systematic reviews (CRD42023406584).

### 2.1 Search strategy and selection criteria

MEDLINE (Medical Literature Analysis and Retrieval System Online) via PubMed, EMBASE (Excerpta Medical Database), and the Cochrane Library were searched using the following search string: ‘Zero Fluoroscopy’ or ‘ZF’ or ‘Near Zero Fluoroscopy’ or ‘Fluoroless’ and ‘Ablation’; and ‘Pediatric’ or ‘Young’ or ‘Children’.

All identified studies were screened by title and abstract. Three investigators independently identified studies that met the inclusion criteria. The study population included patients ≤21 years old who underwent ablation with ZF or near‐ZF with mean fluoroscopy time of ≤1.5 min guided by 3D‐EAM. This cutoff aligns with ALARA recommendations^3^ and is adopted from the adjusted mean fluoroscopy time from near‐ZF reports encountered in literature. Meanwhile, the control group included pediatric patients who underwent conventional fluoroscopy‐guided ablation and/or a mean fluoroscopy time of >1.5 min. Studies were excluded if the subjects were >21 years old and included patient groups with a mean fluoroscopy time of >1.5 min, ZF or near‐ZF guided by echocardiography, or a control group in studies with a fluoroscopy duration of ≤1.5 min.

### 2.2 Data extraction and quality assessment

Standard forms were used to extract the following information from each study: (i) study design and methodology; (ii) type of 3D‐EAM used; (iii) specific characteristics of the intervention and control groups (patient inclusion criteria); (iv) type of ablation (radiofrequency catheter ablation (RFCA) or cryoablation); (v) presence or absence of congenital heart disease (CHD) in the population; (vi) mean fluoroscopy time; (vii) type of arrhythmias ablated; and (viii) effectiveness and safety outcome as stated in the protocol of the current meta‐analysis.

Subsequently, the systematic quality assessment of included studies was evaluated with the preferred Newcastle‐Ottawa Scale (NOS) for observational studies.^15^ Investigations were classified as having low (<5 points), moderate (5–7 points), or high quality (>7 points).

### 2.3 Outcome measurement

We focused on the effectiveness and safety of ZF or near‐ZF as the main clinical outcomes of this study. Short‐term effectiveness or acute success is generally defined as suppression or modulation of the slow pathway in the case of Atrioventricular Nodal Re‐entry Tachycardia (AVNRT), as the absence of inducibility in the case of Atrial Tachycardia (AT), absence of bidirectional conduction after adenosine triphosphate infusion in the case of Accessory Pathway (AP)/Atrioventricular Re‐entry Tachycardia (AVRT),^16^, ^17^ and absence of ventricular arrhythmias under ventricular extra‐stimulation (maximum of three), with or without isoproterenol infusion in the case of Ventricular Tachycardia (VT).^18^ Long‐term effectiveness is determined by arrhythmia recurrence, defined by the recurrence of arrhythmias similar to that identified at the time of catheter ablation. We gauged procedural safety by assessing complications associated with catheter ablation that occurred within 30 days of ablation. Other specific criteria are reported in the results table labelled as study characteristics. The secondary outcomes in this study are the total procedural time and fluoroscopy time.

### 2.4 Data synthesis and analysis quality assessment

We synthesized data that were presented in at least two included studies. Each outcome was analyzed as a subgroup based on the type of zero fluoroscopy. True zero fluoroscopy (true ZF) did not use fluoroscopy at all, and near‐ZF used fluoroscopy with a mean fluoroscopy time of ≤1.5 min. We classified arrhythmias into three subgroups: AVNRT, AVRT, and nonspecified/other arrhythmias, as AVRT and AVNRT were the most frequently studied arrhythmias and several studies did not distinguish between type. These subgroups were compared using the *Z* statistic. Mean and standard deviation (SD) were used to present continuous variables. Sample size, median, range, and quartiles were used to approximate the mean and SD if they were not reported.^19^, ^20^ Subgroup analysis was also performed based on the application of 3D‐EAM and comparison between ZF/NZF with 3D‐EAM and conventional fluoroscopy.

Heterogeneity between study populations was calculated using the *I* ^2^ statistic,^12^ where values of less than 25%, 50%, and 75% were considered evidence of low, medium, and high levels of heterogeneity, respectively. Data were summarized across groups using the Mantel–Haenszel (M–H) risk ratio (RR) fixed‐effect model if *I* ^2^ < 25%. The random‐effect model is used if *I* ^2^ > 25%.^21^ Publication bias was then evaluated using funnel plots and our analysis was carried out using Review Manager 5.4.

## 3 RESULTS

### 3.1 Selection and description of studies

**FIGURE 1:**
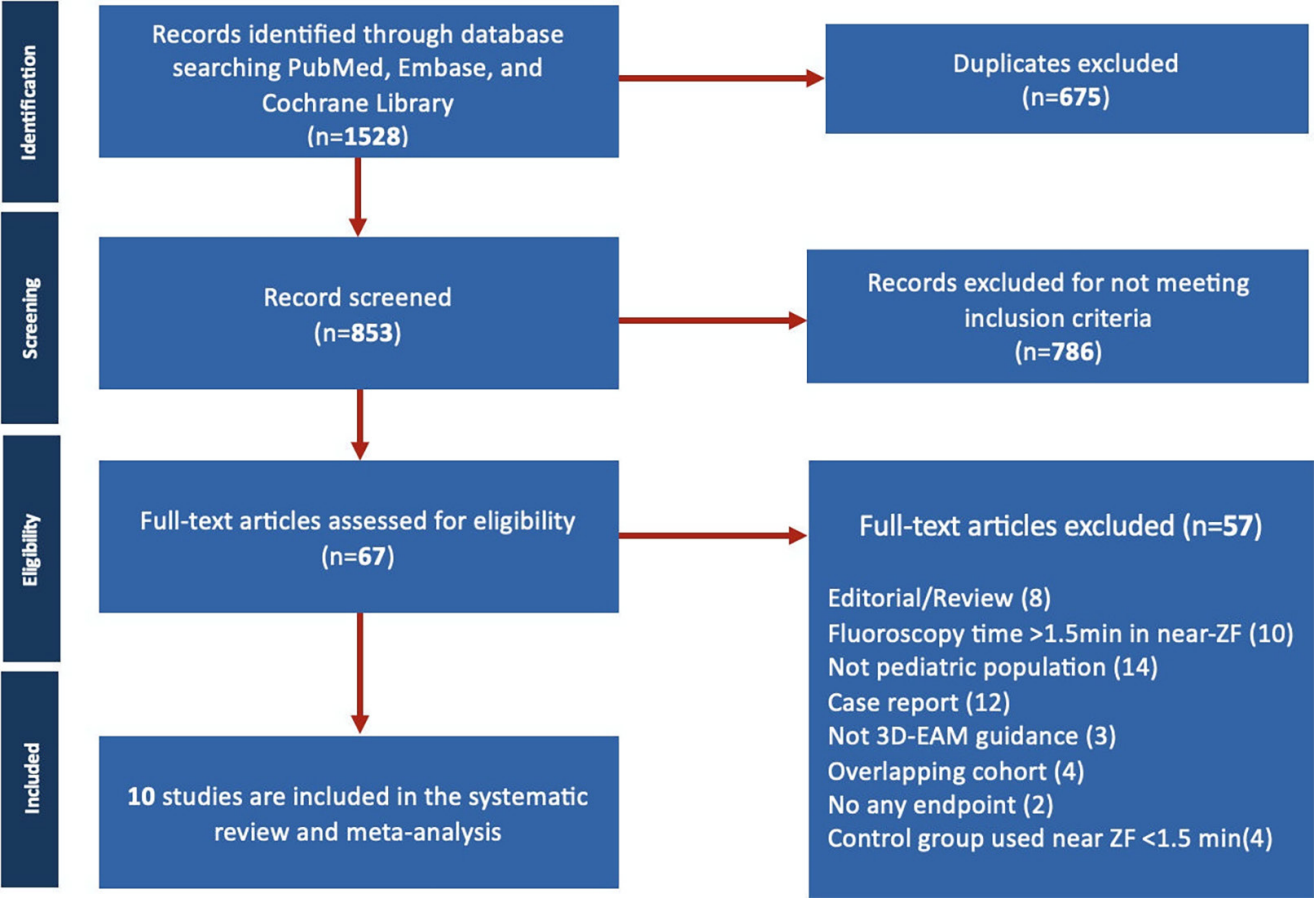
Preferred reporting items for systematic reviews and meta‐analyses (PRISMA) flow diagram. 3D‐EAM, 3D‐electroanatomical mapping; ZF, zero fluoroscopy.

The PRISMA flow diagram in Figure 1 summarizes the study selection process. The primary search identified 1528 citations, and after removing duplicates, the remaining 853 abstracts were independently screened by three researchers. Considering the inclusion criteria, a total of 67 potentially relevant studies were selected for full‐text review and 57 studies were excluded. At the end of this process, 10 cohort studies were included in our data synthesis (Figure 1 and Table 1).^9^, ^18^, ^22^, ^23^, ^24^, ^25^, ^26^, ^27^, ^28^, ^29^

**TABLE 1:** Summary of methodology and ablation details of included studies.

No	First author, year	Study design	Sample size		Description of procedures			
			Study group (*n*)	Control group (*n*)	Study group	Control group	Type of ablation and guidance	Type of arrhythmias (*n*)
1	Anderson, 2021	Cohort Prospective, Multicenter	786	481	Patients from The Catheter Ablation with Reduction or Elimination of Fluoroscopy database from September 2006 through September 2018 selected by matching the enrollment criteria of the Prospective Assessment after Pediatric Cardiac Ablation study, as closely as possible. Exclusion criteria were patients with more than trivial CHD, cardiomyopathy, and patients undergoing any simultaneous non‐EP procedures	Prospective Assessment after Pediatric Cardiac Ablation cohort,^28^ children aged 0–16 years, undergoing catheter ablation for SVT. Exclusion criteria were patients with more than trivial CHD, cardiomyopathy, and patients undergoing any simultaneous non‐EP procedures	RFCA or Cryo Ablation; In study group guided by CARTO (Biosense Webster CV ©) or Ensite (Jude Medical/Abbott ©) without fluoroscopy; In control group guided by conventional fluoroscopy	AVNRT (288) and AVRT (498) (parahisian AP and Non‐parahisian AP)
2	Drago, 2016	Cohort Prospective, Single Center	30	30	Pediatric patients >4 years old and >15 kg with left‐sided APs underwent RFCA	Children matched for age, weight, and tachycardia mechanism, who most recently underwent RFCA of a left‐sided AP at author's Institution using only the CARTO™ 3 system before the era of the UNIVUTM module	RFCA; In study group guided by CARTO UNIVU™ (Biosense‐Webster, Inc.) with ALARA principles were applied when using the fluoroscopy machine; In control group guided by CARTO™ 3 (Biosense‐Webster, Inc.) with ALARA principles were applied when using the fluoroscopy machine	Left‐sided AP (60)
3	Cui, 2022	Cohort Prospective, Single Center	26	20	Children with SVT with (1) successful resuscitation after cardiac arrest because of preexcitation syndrome; (2) accompanied by cardiac dysfunction or recurrent or persistent SVT; (3) symptomatic SVT with body weight ≥ 15 kg or preexcitation cardiomyopathy not responsive to pharmacologic treatment; (4) SVT controllable by pharmacologic therapy and body weight ≥15 kg; (5) pharmacologic therapy for SVT ineffective or intolerable and body weight <15 kg; (6) preexcitation cardiomyopathy with body weight <15 kg and pharmacotherapy ineffective; and (7) preoperative Ebstein's anomaly with preexcitation syndrome and body weight ≥15 kg. Children were excluded if they had CHD, AF or VA, a bleeding tendency, unable to tolerate RFCA or had other contraindications, and if they were unable to attend for follow‐up	Same population as study group	RFCA; In study group guided by CARTO 3 system (Biosense‐Webster, Diamond Bar, CA, United States) without x‐ray fluoroscopy. In control group guided by CARTO 3 system (Biosense‐Webster, Diamond Bar, CA, United States combined with x‐ray fluoroscopy	AVNRT (18), AVRT (27), and AT (1)
4	Maqueda, 2020	Cohort Retrospective, Single Center	62	58	Pediatric population with first or redo ablation with high risk AP or SVT. ZF was planned when the ECG indicated an arrhythmic substrate in the right cavities. Near ZF procedure was planned in patients with asymptomatic preexcitation or clinical palpitations without documented tachycardia	Consecutive series of pediatric patients with high‐risk AP or SVT treated with ablation	RFCA or Cryo Ablation; In study group guided by CARTO3 system (Biosense Webster, United States). In control group guided by fluoroscopy	AVNRT (25), AVRT (6), and AT (89)
5	Rahman, 2021	Cohort Retrospective, Multi Center	55	56	Pediatric population with history of arrhythmia and CHD. Patients who had previous ablations or concurrent diagnostic or interventional catheter procedures and those in whom the precise location of the arrhythmia was not reported were excluded	Matched for operating physician, arrhythmia mechanism, arrhythmia location, weight and age but non‐CHD patients	RFCA; In both group guided by EnSite (St. Jude Medical/Abbott) or CARTO, (Biosense Webster)	AVNRT (22), AP (20), VA (5), Macro‐reentry AT (6), Focal AT (3)
6	Smith, 2007	Cohort Retrospective, Single Center	26	30	The only criterion for inclusion was the presence of reentrant SVT	Pediatric population matched for age and tachycardia mechanism the same hospital registry for patients undergoing the procedure just prior to first use of NavX in 2003. a matched control group required including patients from the database between 2001 and 2003. During this time period we were not yet performing cryoablation	RFCA or Cryo Ablation (Cryo Ablation option was available only in study group); In study group guided by Ensite system (St. Jude Medical, St Paul, MN, USA). For all uncomplicated SVT it is used in the NavX mode (NavX). In control group guided by fluoroscopy	AVNRT (18) and AP (12)
7	Swissa, 2017	Cohort Retrospective, Single Center	64	75	Children with documented SVT, confirmed to be AVNRT in EPS and underwent RFCA of the slow pathway from December 2013 to May 2015 guided by the 3D‐EAM and limited fluoroscopy	Children with documented SVT, confirmed to be AVNRT in EPS and underwent RFCA of the slow pathway from June 2011 to November 2013 guided by the standard fluoroscopy approach	RFCA; In study group guided by EnSite NavX™, (St. Jude Medical, St. Paul, MN, US); In control group guided by standard fluoroscopy techniques	AVNRT (139)
8	Tseng, 2019	Cohort Retrospective, Single Center	41	62	Pediatric population who received ablation procedures for right‐sided AP‐related AVRT and AVNRT under ZF guidance	Pediatric population from who received ablation procedures through conventional fluoroscopy guidance in the same center as study group	RFCA or Cryo Ablation; In study group guided by The combination of IE (EP‐WorkMateTM, St. Jude Medical, West Berlin, NJ, USA), EnSite NavX™ (St. Jude Medical, St. Paul, MN, USA) and CARTO 3 (Biosense Webster), and ICE (ViewFlex Xtra, St. Jude Medical, St. Paul, MN, USA and AcuNav (Biosense Webster); In control group guided by conventional fluoroscopic	AVNRT (47) and right‐sided AP‐related AVRT (56) (parahisian AP and Non‐Parahisian AP)
9	Tseng, 2022	Cohort Retrospective, Single Center	55	48	Pediatric population from January 2012 to June 2020 those who underwent ablation of left‐sided arrhythmia substrates. For those with recurrence of the same arrhythmia substrate, only the first ablative procedure was enrolled	All consecutive patients and framed a historical cohort in the nearest period those undergoing conventional fluoroscopy‐guided procedures between January 2012 and November 2016	RFCA; In study group guided by EnSite Precision (Abbot, St. Paul, MN, USA); In control group guided by conventional fluoroscopy	Left‐sided arrhythmia substrates, including left‐ sided AP‐mediated AVRT (78), and focal or reentrant fascicular VT (18)
10	Tuzcu, 2012	Cohort Retrospective, Single Center	183	93	Younger children, or in some patients with complex CHD	Patients with a left‐sided arrhythmia substrate that need for a transseptal puncture, younger patients in addition to EnSite imaging during navigation of the catheters because of the stiffness of the cryoablation catheters	RFCA and Cryo Ablation; In study group guided by EnSite NavX/Velocity™ systems (St. Jude Medical Inc., St. Paul, MN, USA); In control group guided by EnSite NavX/Velocity™ systems (St. Jude Medical Inc., St. Paul, MN, USA) and conventional fluoroscopy	AVNRT (106), AVRT (156), AT (22), JET (3), VT (15)

Abbreviations: 3D‐EAM, 3D‐Electroanatomical mapping; AF, atrial fibrillation; ALARA, as low as reasonably achievable; AP, accessory pathway; AT, atrial tachycardia; AV, atrioventricular; AVNRT, atrioventricular nodal reentrant tachycardia; AVRT, atrioventricular re‐entry tachycardia; CHD, congenital heart disease;ECG, electrocardiography; EP, Electrophysiology; EPS, electrophysiology study; JET, junctional ectopic tachycardia; RFCA, radiofrequency catheter ablation; SVT, supraventricular tachycardia; VA, ventricular arrhythmia; VT, ventricular tachycardia; ZF, zero fluoroscopy.

### 3.2 Characteristics of included studies

Characteristics of the included studies are presented in Tables 1 and 2. Five studies used a single radio‐frequency catheter ablation (RFCA) strategy for the procedure,^22^, ^23^, ^25^, ^26^, ^27^ while others used either RFCA or cryoablation alone, or a combination of both.^9^, ^23^, ^24^, ^28^, ^29^ In the study group, four studies used true ZF.^18^, ^23^, ^26^, ^28^ One study using near‐ZF in the study group included subgroup analysis data based on true ZF and near‐ZF.^23^ The control group as a whole used conventional fluoroscopy guidance only except for four studies that also used 3D‐EAM guidance.^22^, ^25^, ^26^, ^28^ Reasons for ablation were varied, with the majority being AP/AVRT and AVNRT.

**TABLE 2:** Primary endpoint and baseline characteristics of population in included studies.

No	First author, year	Specific definition and assessment of the primary endpoint	Mean follow‐up time (month)			Demographic data				
			Study group	Control group	All	Preablation AADs management	CHD	Mean age (years)	Mean BMI/BM (kg/m^2^ or kg)	Male (%)
1	Anderson, 2021	Complications: In study group there were transient third degree heart block (*n* = 5), second degree block (*n* = 6), first degree block (*n* = 5), bundle branch block (*n* = 4), hematomas (*n* = 3), severe medication reaction (*n* = 1), bleeding requiring transfusion (*n* = 1) and 3 complications listed as “other”. There was no permanent AV block, radiation‐induced skin burns, or deaths. In the control group, there were permanent heart block (*n* = 6), skin burn (*n* = 1), and intra‐cardiac thrombus (*n* = 1)	NA	NA	NA	NA	Patients with more than trivial CHD were excluded	11.3	21 (BMI)	52.5
2	Drago, 2016	Acute success: Disappearance of ventricular preexcitation or noninducibility of AVRT by programmed stimulation, either at rest or under isoproterenol infusion (0.01–0.04 mg/kg/min) for at least 30 min from the last RF delivery After the procedure, continuous ECG monitoring was maintained for 24 h, and both a surface ECG and a transthoracic echocardiogram were recorded during hospitalization. Clinical evaluation, ECG, 24‐h Holter monitoring, and exercise stress test were performed 1 and 6 months after the procedure	NA	NA	9.1	Antiarrhythmic therapy (not specified). 11 in study group and 16 in control group	NA	10.4	45 (BM)	66.7
3	Cui, 2022	Acute success: In AVNRT after observation for 30 min if atrial and ventricular EP stimulation did not induce AVNRT, the slow pathway conduction disappeared, or residual slow pathway conduction was unaccompanied or only accompanied by a single atrial echo. In AVRT after observation for 30 min if the ECG and pacemaker mapping confirmed blocking of the functions of sequential/reverse transmission of the AP, disappearance of the preexcitation pattern on the surface ECG, and isolation of ventricular pacing or reversal at the AV node. AT was deemed to be treated successfully by RFCA after observation for 30 min if isoproterenol and atrial stimulation did not induce AT Complication: Arteriovenous fistula on the second postoperative day in the control group (*n* = 1). Routine and comprehensive ECG monitoring was performed immediately after the patient returned to the ward. Routine follow‐up was performed at 1, 3, and 6 months in the outpatient department. During the follow‐ up, transesophageal atrial pacing could be performed for patients with frequent symptoms but a normal dynamic ECG to check for recurrence of PSVT	6	6	6	NA	Ventricular septal defect, atrial septal defect, or patent ductus arteriosus were excluded	11.8	18.87 (BMI)	47.8
4	Maqueda, 2020	Acute success: The suppression or modulation of the slow pathway in the case of INT (the presence of up to one nodular echo was accepted), as the absence of inducibility in the case of AT, and as the absence of bidirectional conduction after adenosine triphosphate infusion in the case of AP Complication: first‐degree AV block during RFCA of a medioseptal AP that did not require treatment in study group (*n* = 1), and in control group there was mild pericardial effusion without hemodynamic repercussions or the need for drainage during the ablation of a left posterior hidden AP (*n* = 1) Recurrence: symptomatic recurrence with ECG documentation of tachyarrhythmia or recurrence of ventricular preexcitation Follow‐up visits were made to the hospital of origin: nevertheless, in all cases, one visit per year was made to the pediatric cardiology service of authors hospital	6	6	6	Beta‐blockers, digoxin 22 in study group and 24 in control group. Flecainide, 5 in study group and 9 in control group. Other drugs, 25 in study group and 26 in control group	Bicuspid aortic valve disease (*n* = 2), mitral prolapse (*n* = 2), Ebstein anomaly (*n* = 2), noncompaction cardiomyopathy (*n* = 1), hypertrophic (cardiomyopathy (*n* = 2), corrected single ventricle, and surgically closed subaortic ventricular septal defect (*n* = 2)	11	45.5 (BM)	58.3
5	Rahman, 2021	Acute success: Elimination of tachycardia and all evidence of the mechanism of that tachycardia Complication: transient second degree heart block, transient complete heart block, transient third degree heart block, transient low blood pressure, hematoma and edema, and right external iliac pseudoaneurysm with hematoma	NA	NA	NA	NA	Only in control group (*n* = 56). Consist of Unoperated simple CHD (*n* = 17), repaired two ventricle CHD – excluding TOF and arterial switch (*n* = 15), unoperated acyanotic CHD (*n* = 12), repaired TOF and TOF variants (*n* = 5), prefontan palliated functional single ventricle (*n* = 3), transposition of the great arteries following arterial switch (*n* = 2), unoperated cyanotic CHD (*n* = 1), and fontan palliated functional single ventricle (*n* = 1)	15.05	51.5 (BM)	NA
6	Smith, 2007	Acute success: In patients with AVNRT, success was defined as no inducible SVT either at rest or on Isuprel for greater than 30 min post ablation. Isolated echo beats were acceptable, but not more than single echo beats. For AP‐mediated tachycardia, success was defined as no inducible SVT, either at rest or on Isuprel, for at least 30 min beyond the last lesion	NA	NA	3	NA	No significant CHD	12.6	53.05 (BB)	46%
7	Swissa, 2017	Acute success: Noninducibility of AVNRT, up to a single echo beat. All patients were observed for at least 30 min following the seemingly successful ablation. A complete repeat of EPS with and without isoproterenol was performed postablation in order to ensure success Complication: No procedure related complications All patients were hospitalized overnight for surveillance under continuous rhythm monitoring, and repeated 12‐lead ECG tracing recorded before discharge. Outpatient follow‐up visits were arranged for 1 and 12 months postablation. Recurrence was defined as the return of clinical symptoms or documented SVT	14.3	45.5	NA	NA	All children had a structurally normal heart	12.8	19.8 (BMI)	48.9
8	Tseng, 2019	Acute success: loss of antegrade and retrograde AP conduction, and a lack of tachycardia induction in AVRT. For AVNRT, elimination of the slow pathway with no more than one echo beat was defined as a procedural success Complication: none of the patients experienced major procedural complications requiring emergency or ongoing treatment such as cardiac perforation, pericardial effusion with cardiac tamponade, second or third degree AV block, thrombi, emboli, or death. In addition, none of the patients had vascular complications such as pseudoaneurysm, or arteriovenous fistula during the procedure and follow‐up care for the ablations Recurrency: Documentation of the reappearance of an arrhythmia targeted for ablation	22.7	10.7	NA	Beta‐blockers and others (nonspecified)	Patients with repaired or complex CHD were excluded	13	48.9 (BM)	54
9	Tseng, 2022	Acute success: of both antegrade and retrograde AP conduction and lack of inducible tachycardia (for AVRT) or as absence of VA under ventricular extrastimulation (maximum of three), with or without isoproterenol infusion (for VT) Complication: no major procedural complications, such as cardiac perforation, high‐level AV block, or thromboembolic events, calling for emergency or ongoing treatment; and no deaths occurred. One patient in the study group developed a pseudoaneurysm of right common femoral artery 2 weeks postablation after long‐distance hiking. The patient recovered well by injecting the lesion with thrombin. None of the patients experienced other vascular complications Recurrency: documented reappearance of the same arrhythmia substrates originally ablated at any time points after the ablative procedures Echocardiographic follow‐up was scheduled at 6 months after ablative procedures. For patients who had symptoms, such as dizziness, syncope, shortness of breath, palpitation, or chest pain, a 24‐h Holter or a cardiac event recorder would be performed to detect possible recurrences of arrhythmia	NA	NA	16.7	In the study group, beta‐blockers (*n* = 39), propafenone (*n* = 3) and verapamil (*n* = 2, and combined regimen (*n* = 3), no AADs before the procedures (*n* = 8). After catheter ablation procedures, all AADs were discontinued	Patients with repaired or complex CHD were excluded. None of the patients was have an atrial septal defect or patent foramen ovale	13.9	19 (BMI)	65%
10	Tuzcu, 2012	Acute success: complete resolution of arrhythmia substrates Complication: A patient with an AT focus in the epicardial surface of the left atrial appendage suffered temporary diaphragmatic paralysis. The paralysis resolved within about 6 months following procedure. Another patient developed temporary AV block, which resolved in 1 day following the cryoablation procedure. No other complications were noted The first follow‐up was at 7–10 days and then at 6 months in the majority of the patients. In patients with more complicated arrhythmia substrates or significant CHD, follow‐up was organized at shorter intervals	NA	NA	42.4	NA	Unspecified CHD in study group (*n* = 5) and control group (*n* = 4)	13	53.95 (BM)	62.3%

Abbreviations: 3D‐EAM, 3D‐Electroanatomical mapping; AADs, antiarrhythmic drugs; AF, atrial fibrillation; AP, accessories pathway; ALARA, as low as reasonably achievable; AT, atrial tachycardia; AV, atrioventricular; AVNRT, atrioventricular nodal reentrant tachycardia; AVRT, atrioventricular re‐entry tachycardia; BM, body mass; BMI, body mass index; CHD, congenital heart disease; ECG, electrocardiography; EP, Electrophysiology; EPS, electrophysiology study; JET, junctional ectopic tachycardia; RFCA, radiofrequency catheter ablation; SVT, supraventricular tachycardia; TOF, tetralogy of Fallot; VA, ventricular arrhythmia; VT, ventricular tachycardia; ZF, zero fluoroscopy.

The definitions, specific measurements, and outcome complications for each endpoint are further presented in Table 2. The average follow‐up period in studies that included follow‐up time was 18.6 months and the mean of all population body weight was 49.41 kg. All but one study^25^ reported details of the population with existing CHD. One study completely excluded patients with CHD.^27^ Five studies excluded subjects with significant CHD but did not specify the type of CHD that was included.^18^, ^23^, ^24^, ^26^ The remaining four studies included patients with CHD, including subjects with bicuspid aortic valve, mitral valve prolapse, Ebstein's anomaly, cardiomyopathy, tetralogy of Fallot, repaired CHDs, and CHDs that were not specifically described. One study considered the presence of CHD as an indication for conducting 3D‐EAM‐guided ZF.^22^

### 3.3 Data synthesis

**FIGURE 2:**
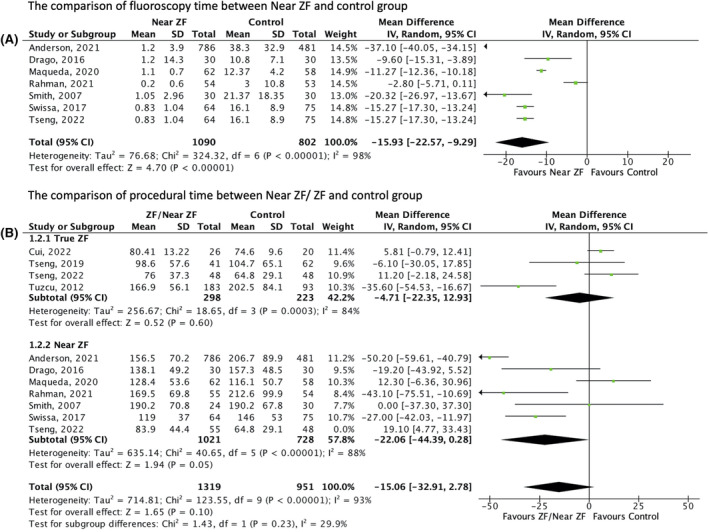
Forest plots of procedural outcomes. (A) A mean difference of fluoroscopy time in near‐ZF compared with the control group. Test for overall effect: *Z* = 76.68 (*p* < .001). Heterogeneity: *I* ^2^ = 98%. (B) The mean difference in procedural time. Test for subgroup differences: *Z* = 1.65 (*p* < .10). Test for overall effect: *Z* = 1.65 (*p* < .10). Overall heterogeneity: *I* ^2^ = 29.9%. CI, 95% confidence interval; RCT, randomized clinical trial; ZF, zero fluoroscopy.

**FIGURE 3:**
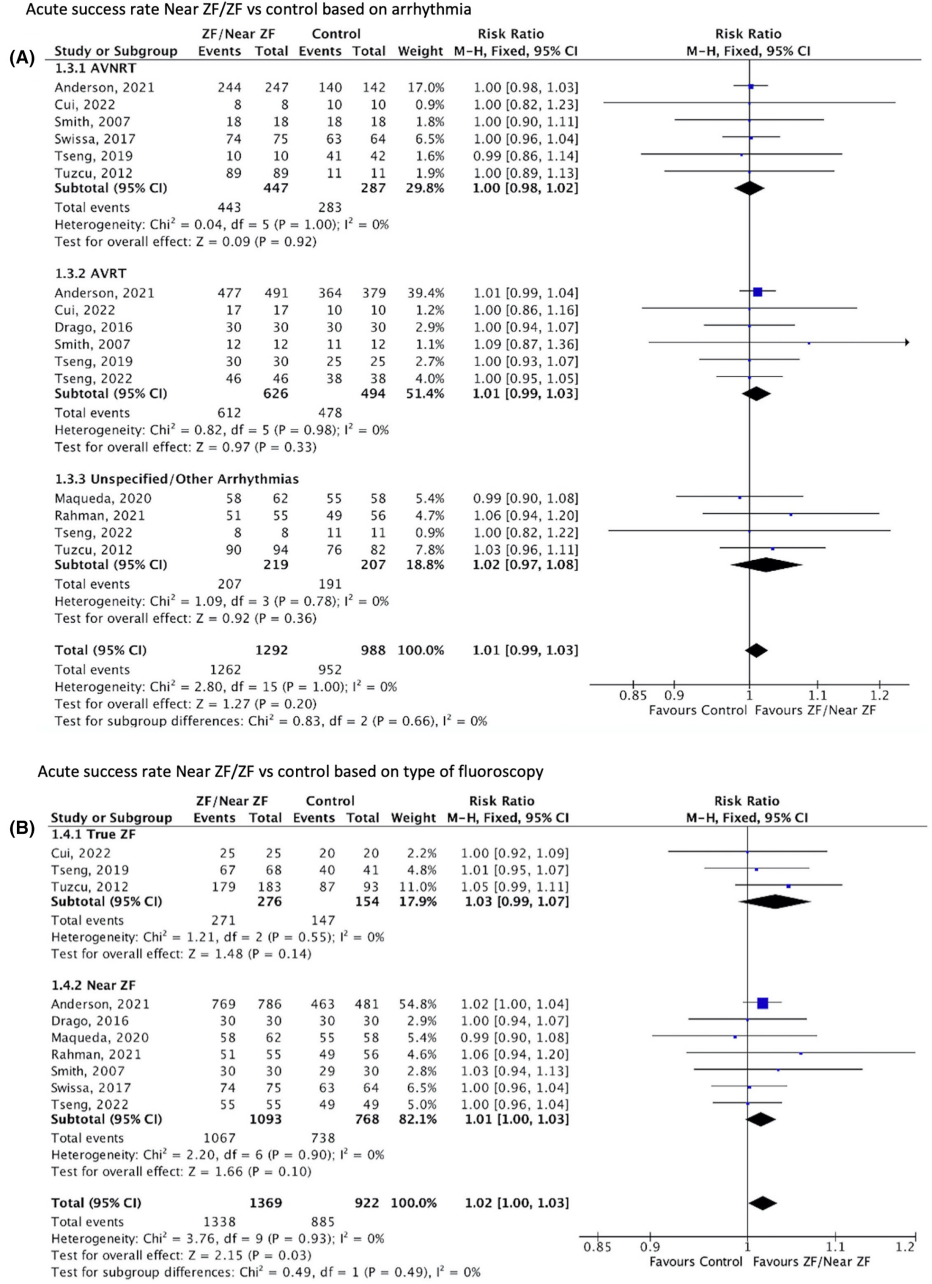
Forest plots of acute success. (A) Risk ratio of acute success based on the type of arrhythmia. Test for subgroup differences: Chi^2^ = 0.83 (*p* = .66). Test for overall effect: *Z* = 1.27 *p* = .20. Overall heterogeneity: *I* ^2^ = 0%. (B) Risk ratio of acute success based on the type of fluoroscopy in the study group. Test for subgroup differences: Chi^2^ = 0.49 (*p* = .49). Test for overall effect: *Z* = 2.15 *p* = .03. Overall heterogeneity: *I* ^2^ = 0%. CI, confidence interval; MH, Mantel‐Haenzel; ZF, zero fluoroscopy. The locations of the forest plot graphs between the controls and the ZF/near‐ZF favors were switched.

**FIGURE 4:**
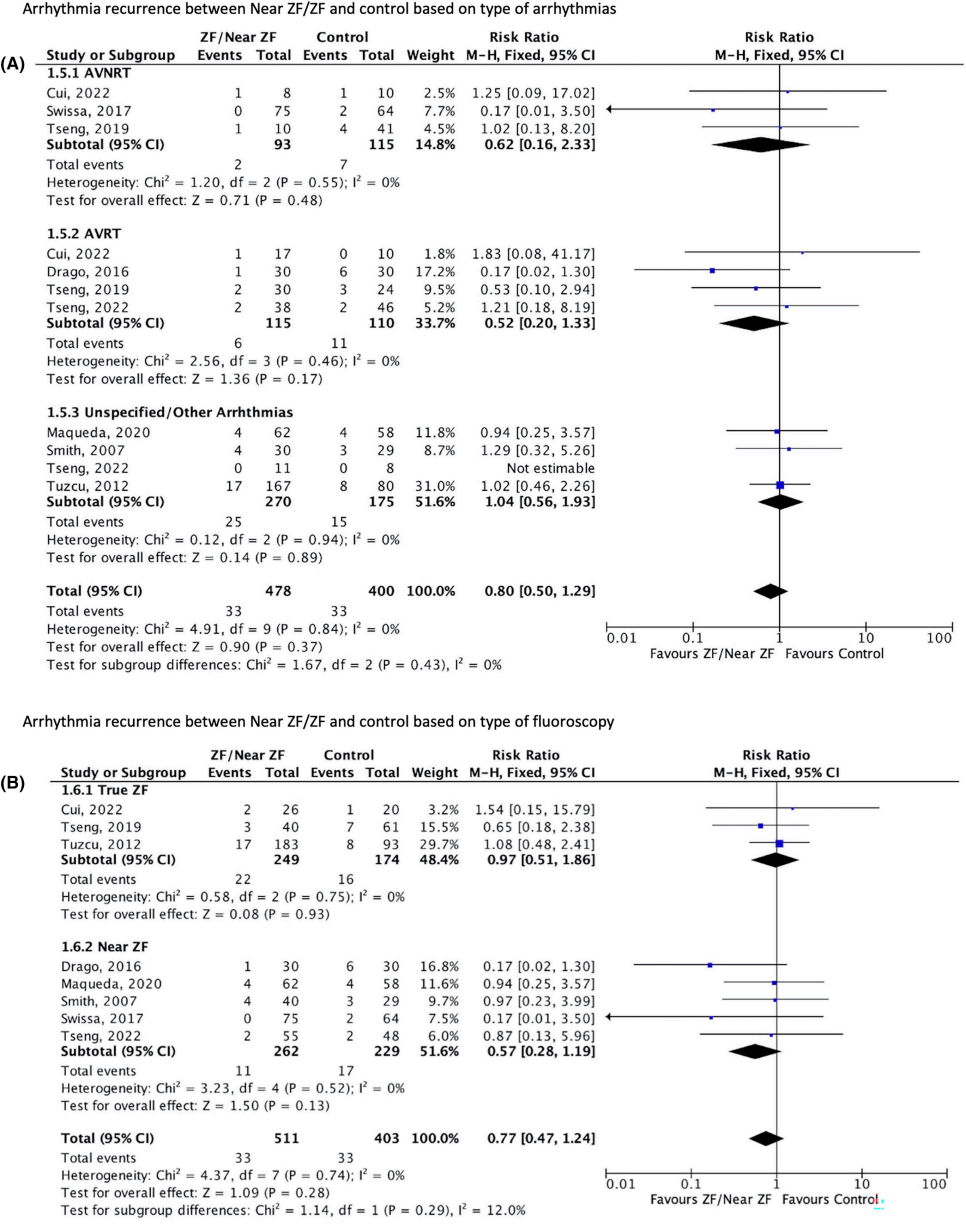
Forest plots of arrhythmia recurrence outcome. (A) Risk ratio of arrhythmia recurrence based on the type of arrhythmia. Test for subgroup differences: Chi^2^ = 1.67 (*p* = .43). Test for overall effect: *Z* = 0.90 (*p* = .43). Overall heterogeneity: *I* ^2^ = 0%. (B) Risk ratio of arrhythmia recurrence based on the type of fluoroscopy in the study group. Test for subgroup differences: Chi^2^ = 1.09 (*p* = .28). Overall heterogeneity: *I* ^2^ = 12.0%. CI, confidence interval; MH, Mantel‐Haenzel; ZF, zero fluoroscopy.

**FIGURE 5:**
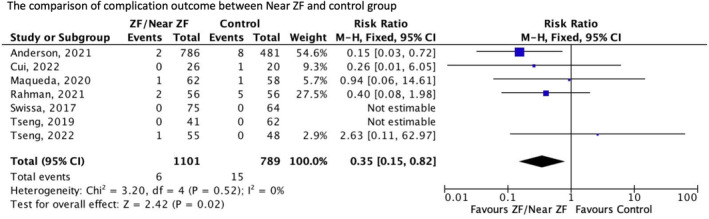
Forest plots of complication outcome. Test for overall effect: *Z* = 2.19 (*p* = .03). Heterogeneity: *I* ^2^ = 0%. CI, confidence interval; MH, Mantel‐Haenzel; ZF, zero fluoroscopy.

The 10 studies comprised of 2279 patients were included in this study with a mean age of 12.7 years and predominately of the male gender (55.7%) (Table 2). The results of the analysis showed that the total fluoroscopy time was significantly lower in the near‐ZF group [MD –15.93 min; 95% CI (−22.57 – (−9.29), *p* < .001; *I* ^2^ = 98%), Figure 2A]. Additionally, the near‐ZF group had a shorter total procedure time [MD –15.06 min; 95% CI (−32.91 – (−2.78), *p* = .05; *I* ^2^ = 88%), Figure 2B] but there was no significant difference on subgroup analysis compared to true ZF, which does not utilize fluoroscopy (*p* = .60). The effectiveness of ZF or near‐ZF showed that in terms of the type of arrhythmia, ZF or near‐ZF had an overall acute success comparable to conventional fluoroscopy **[**Figure 3A **]**. However, true ZF and near‐ZF included in our analysis demonstrated that the overall acute success was significantly better compared to ablation using conventional fluoroscopy [RR 1.02; 95% CI (1.00–1.03), *p* = 0.03; *I* ^2^ = 0%, Figure 3B]. All patients with successful ablation discontinued antiarrhythmic drugs unless a relapse occurred during follow‐up. There was a tendency towards a decrease in arrhythmia recurrence, regardless of the type of arrhythmia [RR 0.80; 95% CI (0.05–1.29), *p* = 0.37; *I* ^2^ = 0%, Figure 4A], as well as the fluoroscopy dose (true ZF, near ZF, conventional fluoroscopy) [RR 0.77; 95% CI (0.47–1.24), *p* = .28; *I* ^2^ = 0%, Figure 4B]. 3D‐EAM‐guided ZF or near‐ZF has a safety profile superior to conventional fluoroscopy [RR 0.35; 95% CI (0.14–0.90); *p* = .03; *I* ^2^ = 0%, Figure 5]. The funnel plot does not show significant publication bias in terms of effectiveness and safety (Supplementary Information S5). In order to reduce the heterogeneity bias from control group, subgroup analysis was conducted (Supplementary Information S6), and was focused on 3D‐EAM utilization on both groups, which the results were 3D‐EAM‐guided ZF or near ZF also shows noninferiority outcome in acute success rate [RR 1.01; 95% CI (1.00–1.03); *p* = .15; *I* ^2^ = 0%], arrhythmia recurrence [RR 0.73; 95% CI (0.37–1.46); *p* = .37; *I* ^2^ = 0%], and complication occurrence [RR 0.46; 95% CI (0.08–2.58); *p* = .38; *I* ^2^ = 36%] when compared to conventional fluoroscopy with 3D‐EAM application.

### 3.4 Risk of bias

The overall quality of included studies ranged from good to moderate, with NOS values ranging from 6 to 9 (Supplementary Information S1). Two studies categorized as having moderate quality omitted specifying the follow‐up duration.^22^, ^24^ All observational studies obtained their data directly from medical records; however, two studies had the disadvantage of employing suboptimal control groups. Their subjects came from different communities or CHD status was taken into account to consider control variables, which has the potential to introduce confounding variables.^22^, ^28^ As previously explained, four studies performed ablation with 3D‐EAM guidance in the control group. We did not exclude these studies as our aim focused more on the outcome comparison between ZF/near‐ZF and conventional fluoroscopy ablation. As additional data, we conducted a sub‐analysis of primary outcomes, specifically in studies using 3D‐EAM in both study and control groups (Supplementary Information S2‐S4).

## 4 DISCUSSION

We found that 3D‐EAM ZF or near‐ZF‐guided ablation in the pediatric population was as effective in both the short and long term compared to conventional fluoroscopy‐guided ablation. Additionally, this technique was associated with a lower risk of complications and a shorter duration of fluoroscopy. However, the true ZF group was not linked with a shorter overall procedural time. Given the many benefits provided by 3D‐EAM‐guided ZF or near‐ZF ablation, this study challenges the need for routine fluoroscopy in the pediatric population, even for short durations and suggests that the 3D‐EAM‐guided ZF ablation strategy should be the technique of choice for ablation of the two most common substrates (AVNRT and AVRT) in the pediatric population, at least in the absence of existing structural heart disease.

In its early days, RFCA in the pediatric population reported significant x‐ray exposure.^30^, ^31^ Today, nonfluoroscopic imaging modalities are widely available, and the reduction of radiation exposure during ablation, especially in children, is considered a critical issue, as underlined by the principle of ALARA.^32^ The pediatric population naturally has a greater life expectancy compared to the general population, and when paired with their rapid physiological somatic growth, augments their vulnerability to the stochastic effects of radiation.^18^ In 2002, Drago et al. first described how radiation exposure could be substantially reduced by using electro‐anatomical mapping systems to carry out ablation in the pediatric population, as well as to help limit the number of catheters utilized.^17^ Besides the side effect of radiation, fluoroscopy can cause skin reddening especially when repeated procedures with the same skin area and hair loss. Further uses will also increase cancer risk and contract‐induced nephropathy (CIN).^33^ More recent studies provide further evidence towards the success of 3D‐EAM‐guided catheter ablation of different arrhythmogenic substrates with minimal or no fluoroscopy.^9^, ^18^, ^22^, ^23^, ^24^, ^25^, ^26^, ^27^, ^28^, ^29^ Unfortunately, ablation in the pediatric population has not been established as a routine treatment modality. In many centers, pharmacological strategies using antiarrhythmic drugs remains the first choice, even in cases of recurrent arrhythmias despite the failure of one antiarrhythmic drug. Catheter ablation is only indicated once two or more antiarrhythmic drugs fail or tachycardiomyopathy is suspected,^31^ even though various studies have shown that early intervention for arrhythmia has proven to provide good long‐term outcomes in the pediatric population, even before the age of one.^28^, ^31^, ^34^, ^35^, ^36^, ^37^ Based on 2016 PACES‐HRS Catheter ablation guideline, catheter ablation is recommended for documented SVT, even it is recurrent or persistent and preferred by the family to avoid long‐term antiarrhythmic medication consumption.^38^ Another reason for not routine treatment is because of the high cost needed in electrophysiology lab because of the high manufacturing cost and we have assumption that the prices will not drop. However, the costs are not standardized between institutions and agencies, so positive effect of CA can be given if the children and teenagers have the higher statistical life values correction factors. A positive effect of value statistical life especially in child's life will give a rise correction factors in children and cause high cost‐effectiveness.^39^

Previous meta‐analyses in 2016 and 2022 included 2261 and 9074 patients, respectively and compared ZF or near‐ZF to fluoroscopic approaches during cardiac arrhythmia ablation.^40^, ^41^ Consistent with the findings of this study, the meta‐analyses also demonstrated significant reductions in fluoroscopy and ablation time, whereas acute and long‐term success rates were similar between the two groups. Our study involved a more homogeneous population, namely the pediatric population, with outcomes that turned out to be slightly different from those of these studies.

In interpreting the study results, we have emphasized the influence of utilizing 3D‐EAM, which appears to play a dominant role in the outcomes of ablation compared to the use of ZF or NZF alone without 3D‐EAM support. Despite the short fluoroscopy time, it is essential to acknowledge the potential contribution of 3D‐EAM to the results. To address this concern, we conducted subgroup analysis comparing cases using 3D‐EAM (all baseline cases included 3D‐EAM) with nonsignificant results, indicating that the focus indeed lies on the utilization of 3D‐EAM, which is shown in Supplementary Information S6.

### 4.1 Procedural parameters

Although the dose threshold for safe radiation is not clearly defined, the subset of subjects we included in this study was exposed to zero or near‐zero fluoroscopy, meaning they were unlikely to result in unwanted long‐term stochastic effects of radiation.^25^ Theoretically, three‐dimensional visualization with the 3D‐EAM system reduces the procedural duration and allows for easier catheter manipulation. However, the 3D‐EAM system requires several minutes to create, which takes longer than conventional methods of fluoroscopy.^41^ Previous meta‐analyses reveal no differences in the procedural time between groups. However, our study found that the overall procedural time in both the combined ZF and near‐ZF group, and the near‐ZF subgroup alone were significantly shorter compared to conventional fluoroscopy. However, *I* ^2^ analysis revealed substantial heterogeneity in our findings. One reason is that subgroup analysis was not carried out for the type of arrhythmia but was performed for other outcomes. Additionally, procedural outcome is greatly influenced by the skills of the operator and standard operating procedures, which differs from center to center.^27^, ^42^ It is uncommon to find arterial or valvular diseases in the pediatric population, such as peripheral arterial occlusive disease or aortic stenosis, that could potentially interfere with procedures. For this reason, establishing arterial access through the aortic valve is much easier in children compared to adults. Additionally, operators may experience lag time from catheter manipulation to visualization on the 3D‐EAM screen. Upon crossing the aortic valve, curvature of the ablation catheter demands additional dexterity to advance to the ideal ablation site. This is of minimal concern with respect to right‐sided substrates, which bypasses the aortic conundrum and explains why Tseng's study showed unique results, as his study was the only one to solely focus on left‐sided substrate ablation.^18^ Transeptal puncture from right atrium to left atrium may also contribute to time consuming. This technique combined with 3D‐EAM and contributes towards a zero fluoroscopy will help in visualizing the electrode tip of round needle, which may not affect the procedural time or outcomes.^43^ In the true ZF subgroup, limited evidence may be the reason there was no significant difference in procedural time. Two of the four studies specified reasons for a shorter procedural time in the control group. One study involved procedures focused on left‐sided substrates and another combined 3D‐EAM with conventional fluoroscopy in the control group. These two reasons, along with limited available evidence, resulted in statistically insignificant results.^18^, ^26^

### 4.2 Effectiveness

The overall acute success rate in the study group was 98%, while that in the control group was 96%. Although both had relatively good success rates, the difference was statistically significant. Despite promising outcomes for acute success, we were unable to prove long‐term success because of the lack of statistical significance. More subgroup analyses involving large data sets are needed to identify factors that may demonstrate statistically significant improvement in long‐term outcomes, such as the type of ablation, type and mode of 3D‐EAM, presence of absence of CHD, and more specific classification of arrhythmia, among other factors.

The acute success rate of AP ablation of the left ventricular free wall is reported to be the highest among all Aps, and has similar outcomes when compared to conventional fluoroscopy, whereas AP ablation of the right ventricular free wall has the lowest success rate and a high rate of recurrence.^44^, ^45^ The use of 3D‐EAM guidance in areas close to the insertion pathway precisely locates and marks atrial and ventricular AP insertion sites, and proves a superior technique when compared to conventional methods. We believe that this explains the positive findings in the study group for acute success rate as majority of interventions involve substrates that would benefit from the use of 3D‐EAM. Additional advantages of 3D‐EAM includes improving catheter stability (e.g., right ventricular free wall passage), repositioning after postablation catheter dislodgement (e.g., termination because of tachycardia), and ease in consecutive mapping of different anatomic locations, especially in the pediatric population.^44^, ^46^, ^47^, ^48^, ^49^, ^50^ Regardless of the cause, our findings confirm that ZF or near‐ZF guiding during ablation in the pediatric population is at least as effective as traditional fluoroscopically guided procedures. None of the trials demonstrated inferior effectiveness outcomes in the study group.

### 4.3 Complications

Although the overall complication rates were low, they were significantly lower in the study group (0.5% and 1.9%, respectively), with no heterogeneity in these outcomes. We considered this finding somewhat interesting as previous meta‐analyses conducted in the adult population found no significant difference in complication rates between the two groups because of the small sample size that was included in this study. Although the complications reported in this study varied, the most common was AV block. This may have resulted from the unique cardiac anatomy of pediatric patients, where the coronary sinus and sub‐eustachian isthmuses are smaller. The compact AV node is more likely to be injured in children because it is situated close to the posterior septal region.^51^, ^52^ 3D‐EAM‐guided ZF or near‐ZF ablation may provide additional benefits in preventing development of AV block by providing continuous visualization of the catheter during the procedure as well as providing tools to mark crucial conduction system landmarks that remain on the image even after the catheter has been removed.^24^

### 4.4 Limitations

This study has the typical limitations of a systematic review and meta‐analysis. All included studies were observational and, although generally of good quality, they are not a substitute for large‐scale RCTs. However, our results were generally consistent across all cohorts, which adds to the robustness of our findings. Learning curve effects cannot be ruled out for nonrandomized studies as several included studies observed findings during 3D‐EAM's early days. However, this appears to have little effect on the main endpoint as it shows consistent effectiveness and safety outcomes across studies with very low heterogeneity. Important differences in patient demographics and procedures that may influence the outcome and were not accounted for in this analysis include infancy (0–12 months of age), body mass index, type of energy in ablation, type and mode of 3D‐EAM, as well as specific types of arrhythmias. Additionally, data limitation on radiation exposure would impact the use of fluoroscopy time which affect the safety and complication in this study.

## 5 CONCLUSION

The first meta‐analysis to evaluate the outcomes of 3D‐EAM‐guided ZF or near‐ZF ablation strategy in the pediatric population showed similar, if not better outcomes compared to conventional fluoroscopy, along with the added benefits of a reduction in radiation exposure and possibly more time‐effective procedures. This strategy should be considered first line with regards to ablation procedures in the pediatric population.

## CONFLICT OF INTEREST STATEMENT

Authors declare no conflict of interests for this article.

## ETHICS STATEMENT

No human participant was involved in this study. The protocol is registered in the international prospective register of systematic reviews (CRD42023406584).

## Supporting information


**Data S1:** Supporting Information.

## Data Availability

All data will be provided in this manuscript and supplementary material.
