# Supplementary material for: Safety and feasibility of 3D‐electroanatomical mapping‐guided zero or near‐zero fluoroscopy catheter ablation for pediatric arrhythmias: Meta‐analysis
Source: J Arrhythm. 2024 May 16;40(4):913–34. doi: 10.1002/joa3.13062 (PMC11317687; doi:10.1002/joa3.13062)

**Supplementary Material**

| **Study** | **Selection** | | | | **Comparability** | | **Outcome** | | | **Total** |
| --- | --- | --- | --- | --- | --- | --- | --- | --- | --- | --- |
|  | **1** | **2** | **3** | **4** | **1** | **2** | **1** | **2** | **3** |  |
| Anderson, 2021 | * | - | * | ***** | * | * | * | - | - | 6 |
| Drago, 2016 | * | * | * | * | * | * | * | * | * | 9 |
| Cui, 2022 | * | * | * | * | * | * | * | * | * | 9 |
| Maqueda, 2020 | * | * | * | * | * | * | * | * | * | 9 |
| Rahman, 2021 | * | * | * | * | * | * | * | **-** | **-** | 7 |
| Smith, 2007 | * | * | * | * | * | * | * | * | * | 9 |
| Swissa, 2017 | * | * | * | * | * | * | * | * | * | 9 |
| Tseng, 2019 | * | * | * | * | * | * | * | * | * | 9 |
| Tseng, 2022 | * | * | * | * | * | * | * | * | * | 9 |
| Tuzcu, 2012 | * | * | * | * | * | * | * | * | * | 9 |

Supplementary Information 1: Risk of bias assessment of observational studies included in the meta-analysis according to the Newcastle-Ottawa Scale.

Supplementary Information 2: (A) Forest plot of acute success in 3D-EAM studies


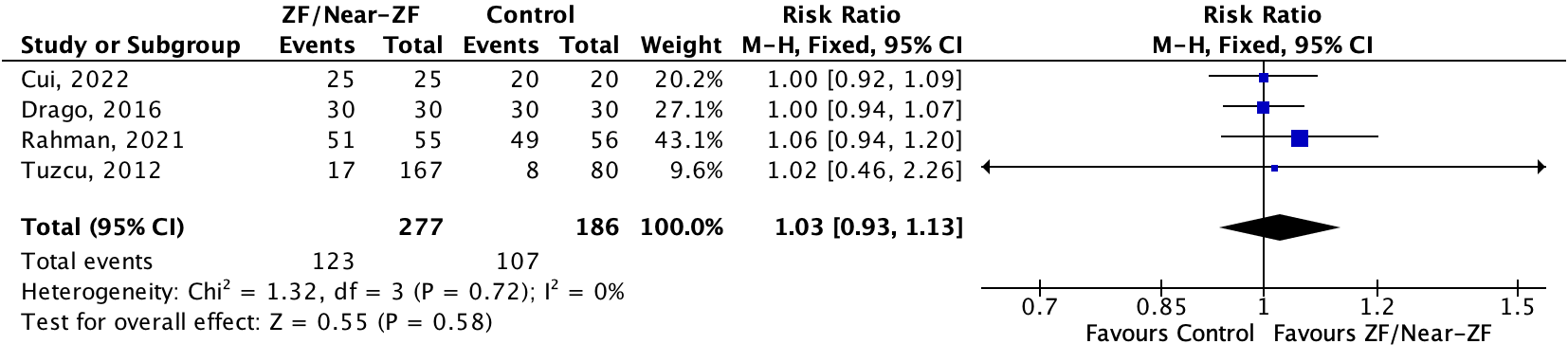


(B) Funnel plot of acute success in 3D-EAM studies


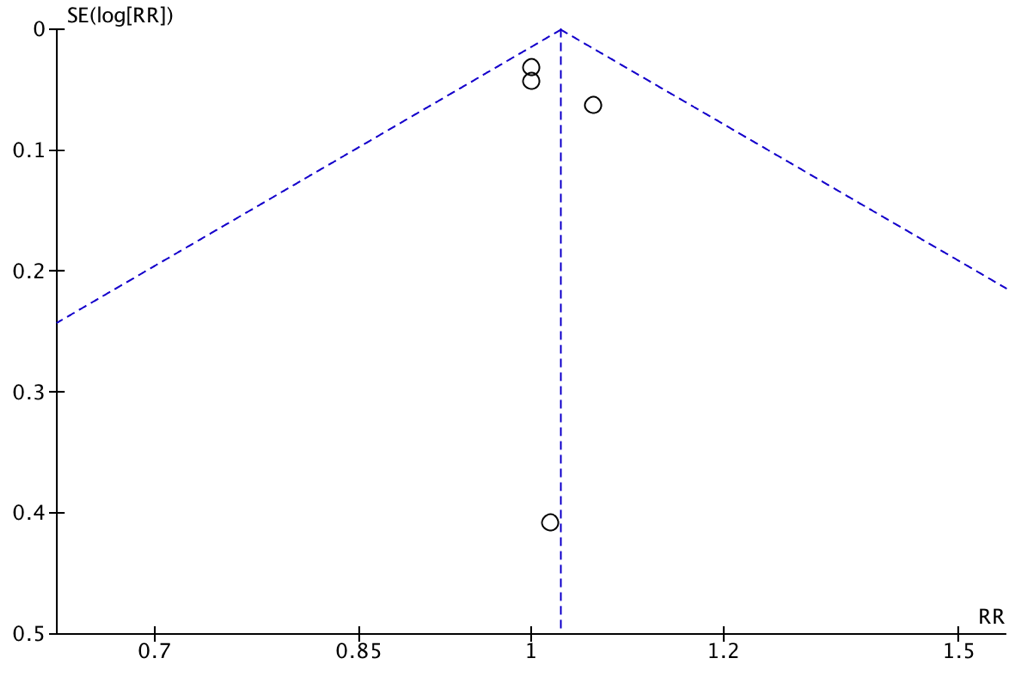


Supplementary Information 3: (A) Forest plot of arrhythmia recurrency in 3D-EAM studies


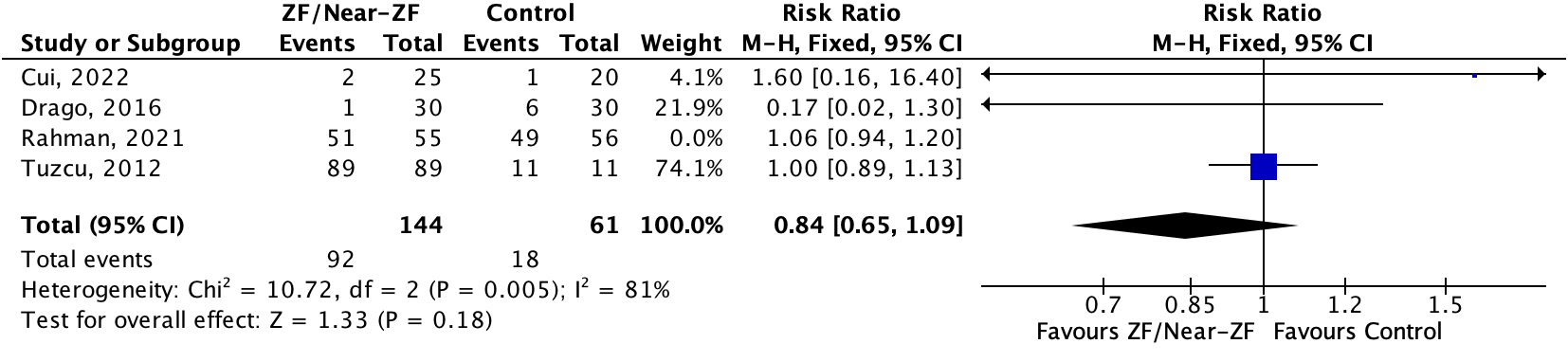


(B) Funnel plot of arrhythmia recurrency in 3D-EAM studies


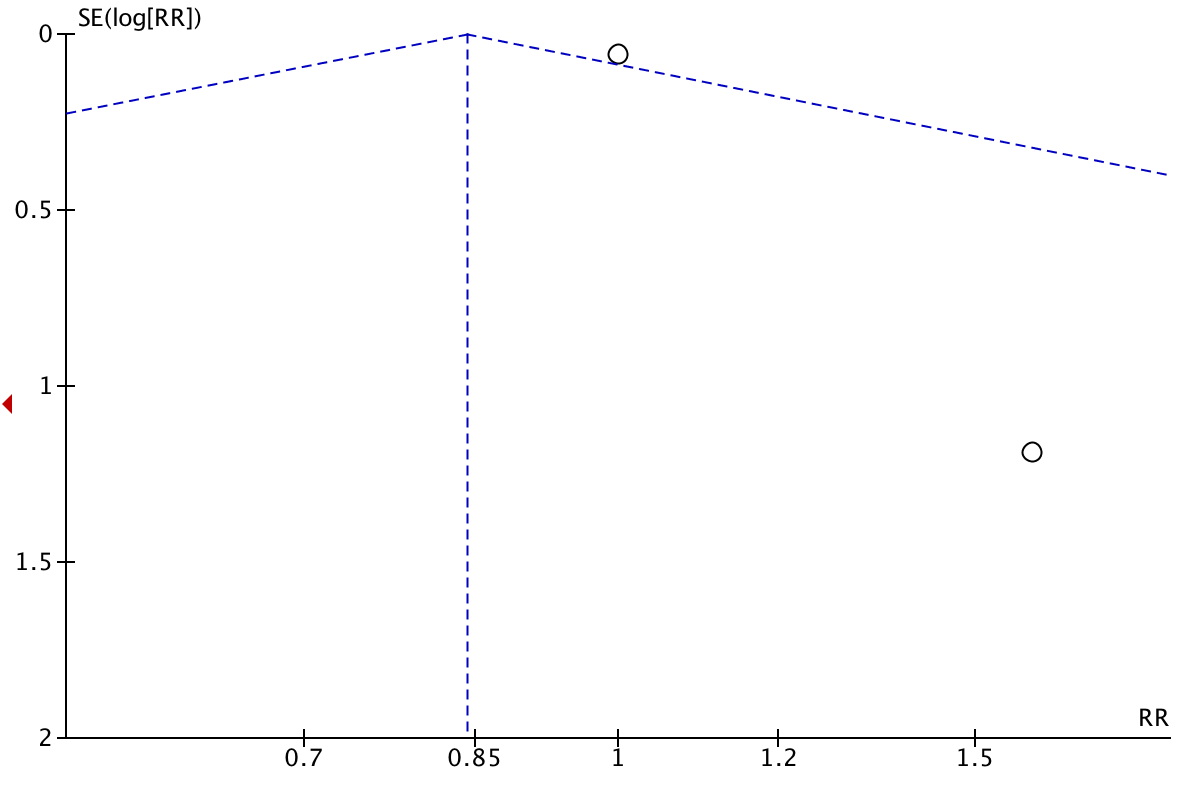


Supplementary Information 4: (A) Forest plot of complication in 3D-EAM studies


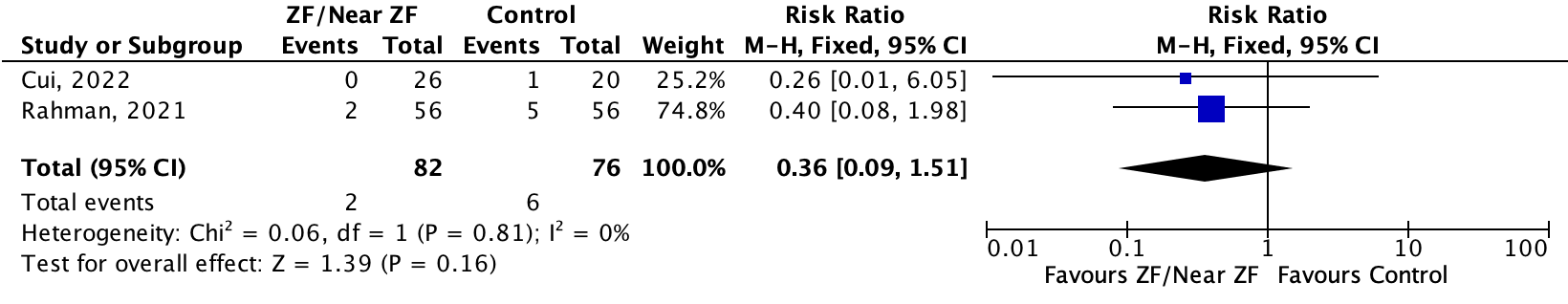


(B) Funnel plot of arrhytmia recurrency in 3D-EAM studies


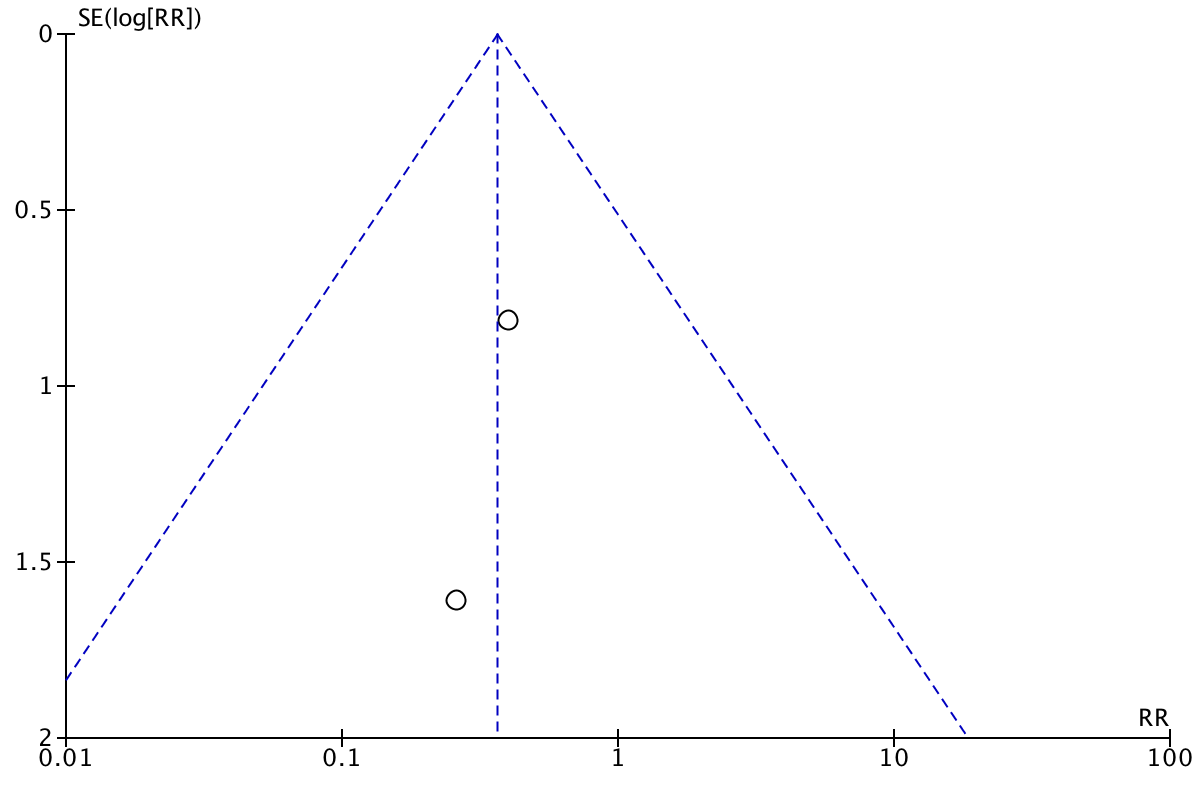


Supplementary Information 5: (A) Funnel plot for fluoroscopy time


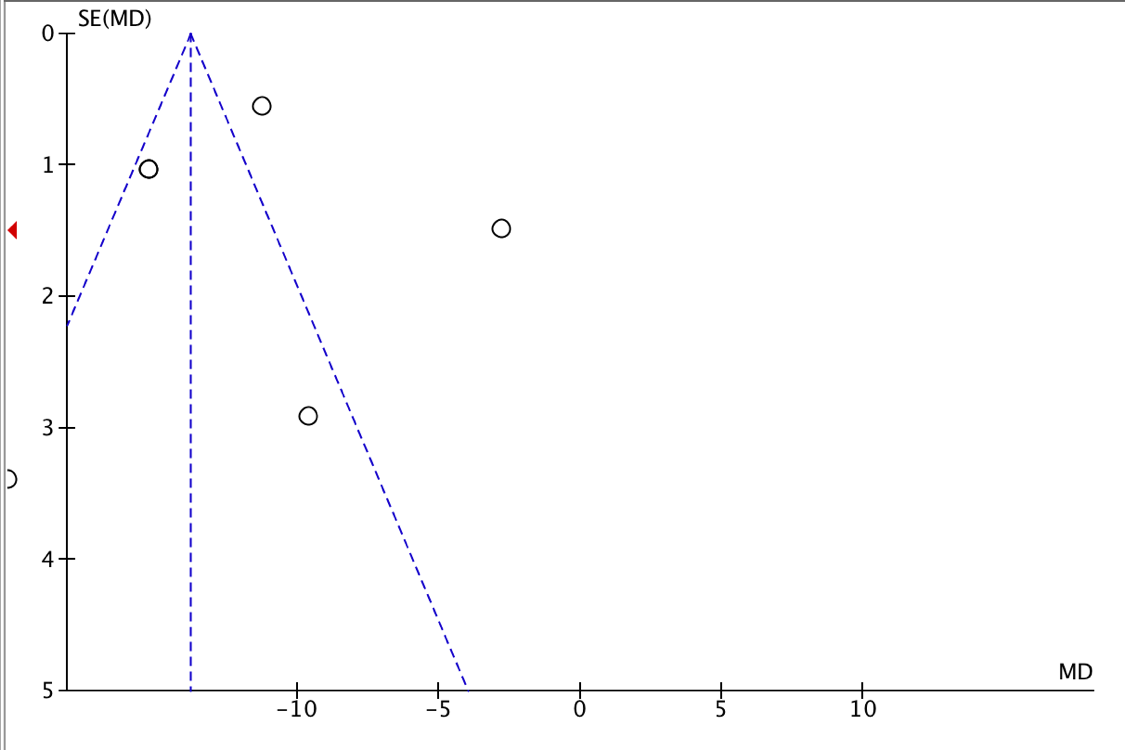


(B) Funnel plot for total procedural time
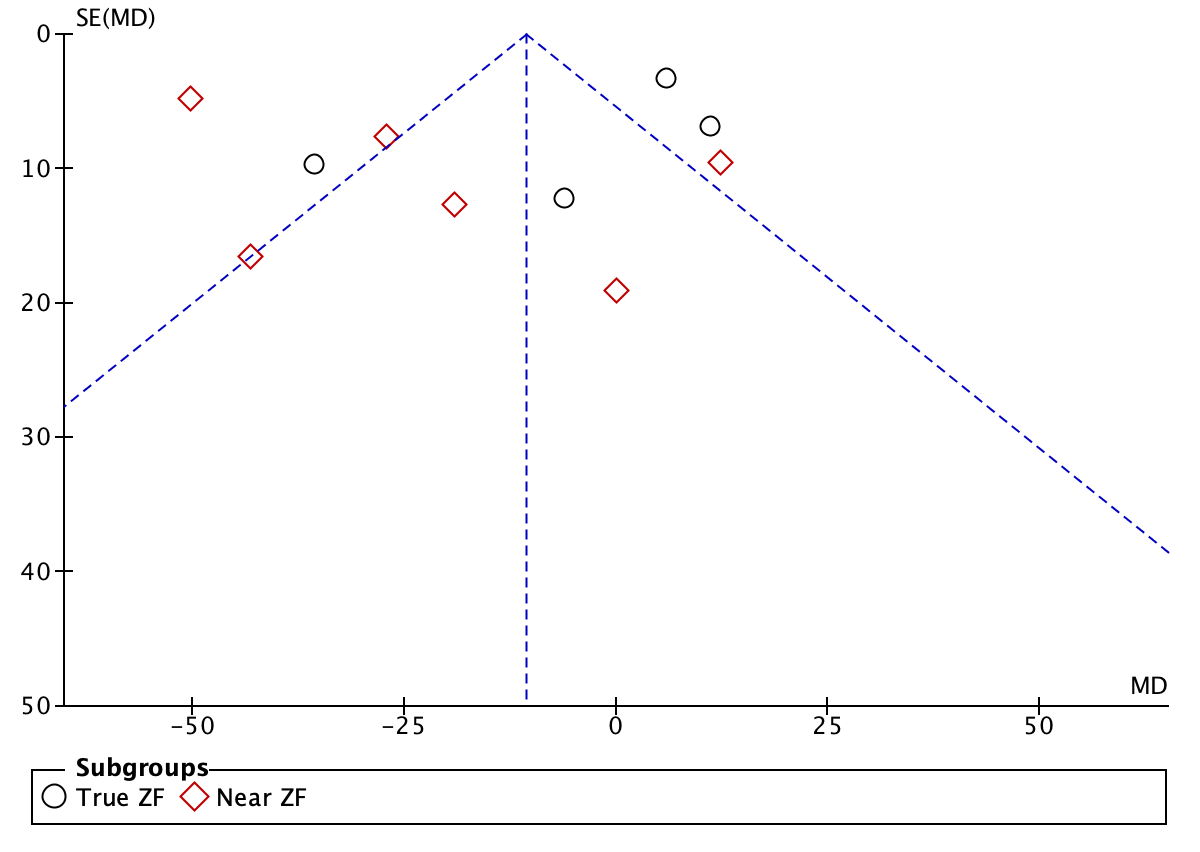


(C) Funnel plot for acute success based on arrhythmia type


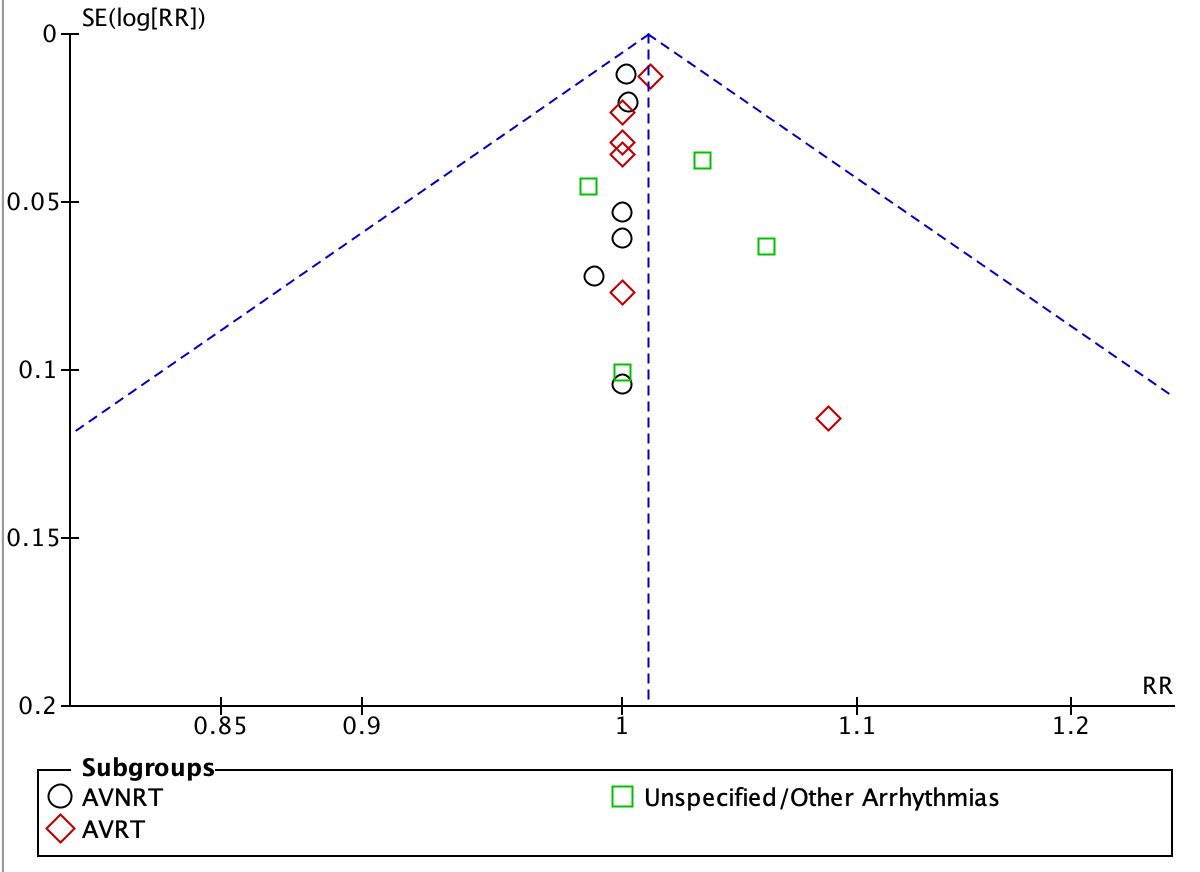


(D) Funnel plot for acute success based on fluoroscopy type


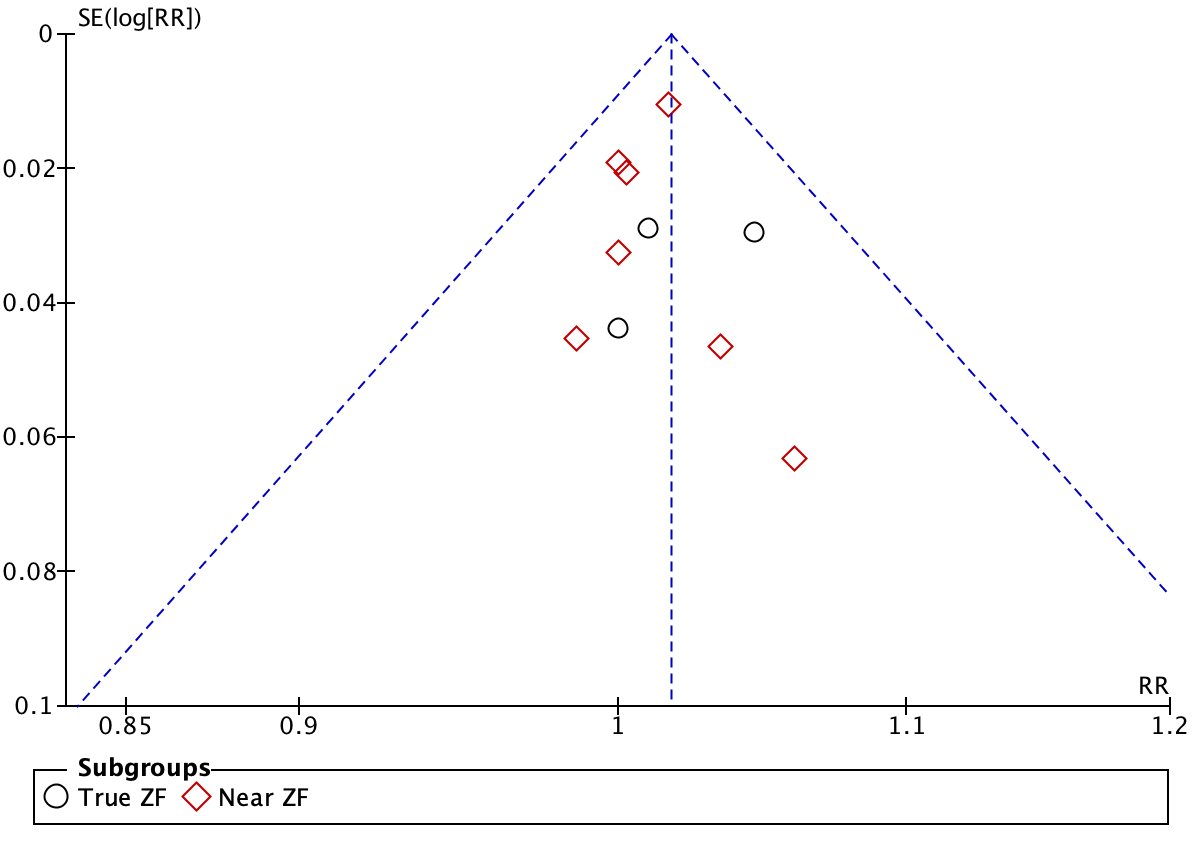


(E) Funnel plot for arrhythmia recurrence based on arrhythmia type


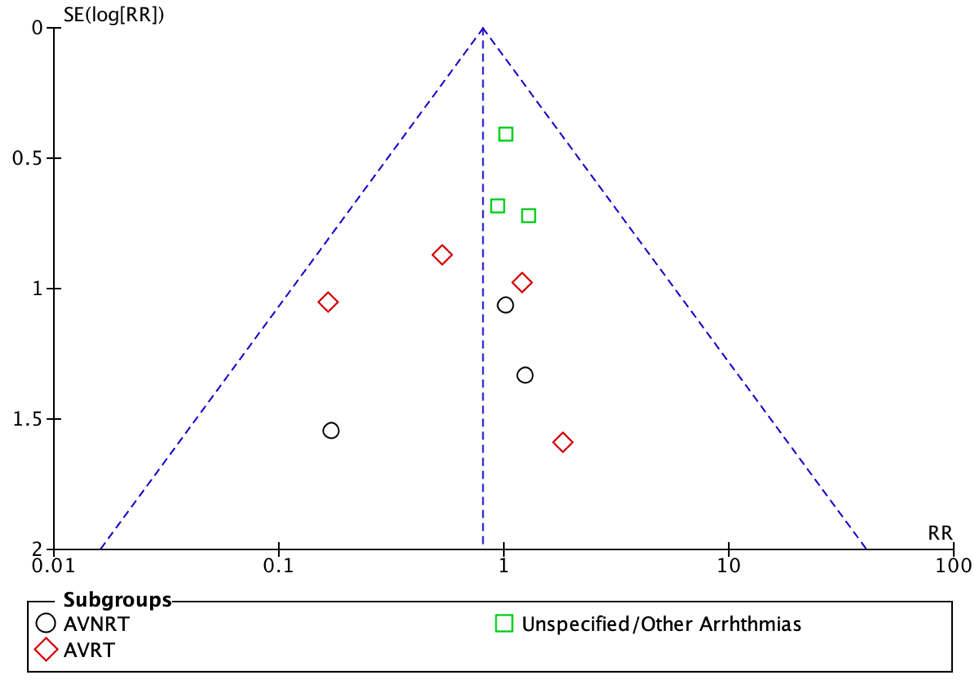


(F) Funnel plot for arrhythmia recurrence based on fluoroscopy type


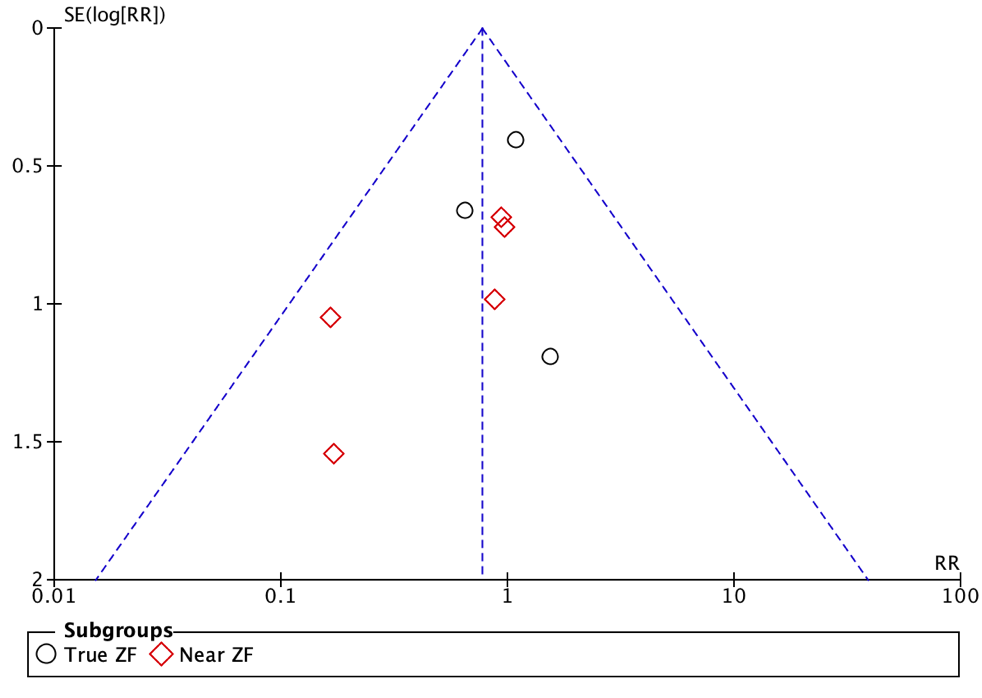


(G) Funnel plot for procedural complication


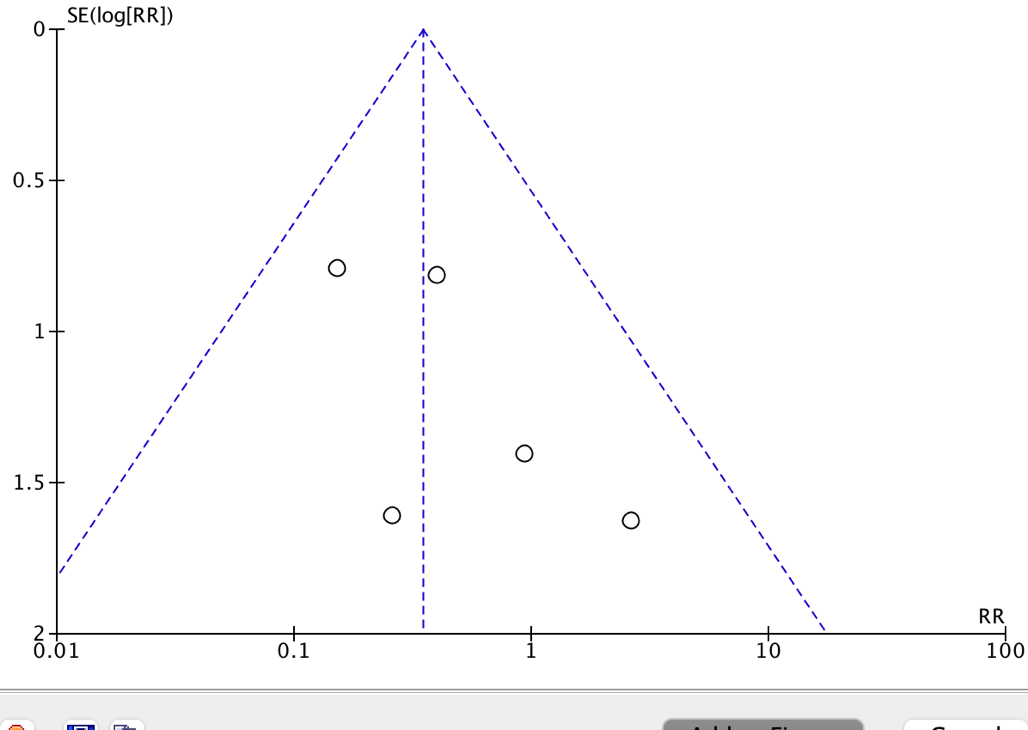


Supplementary Information 6. (A) Forrest plot of acute success rate based on 3D-EAM application


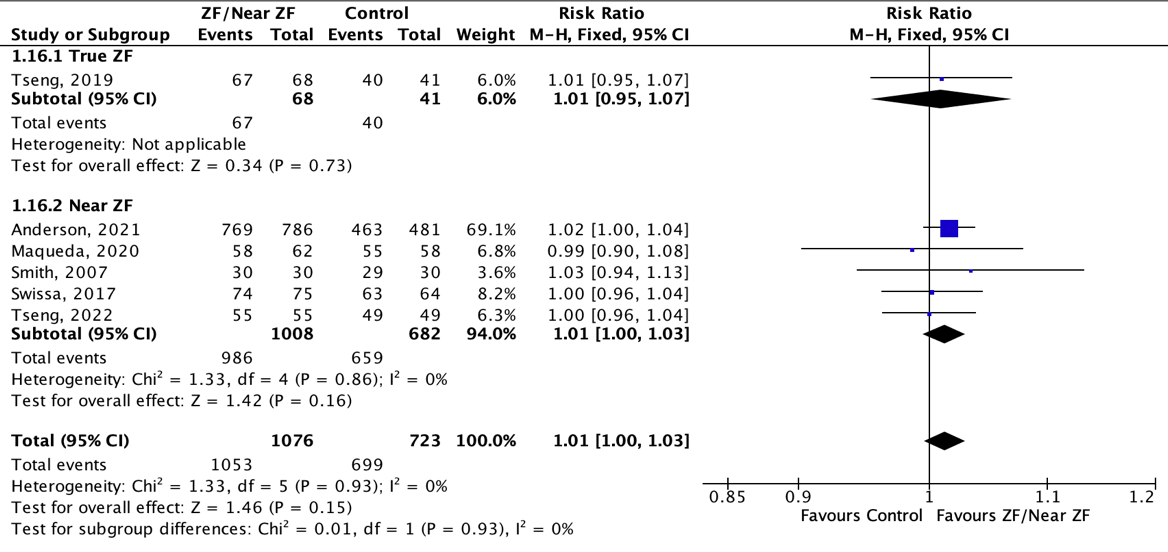


(B) Funnel plot of acute success rate based on 3D-EAM application


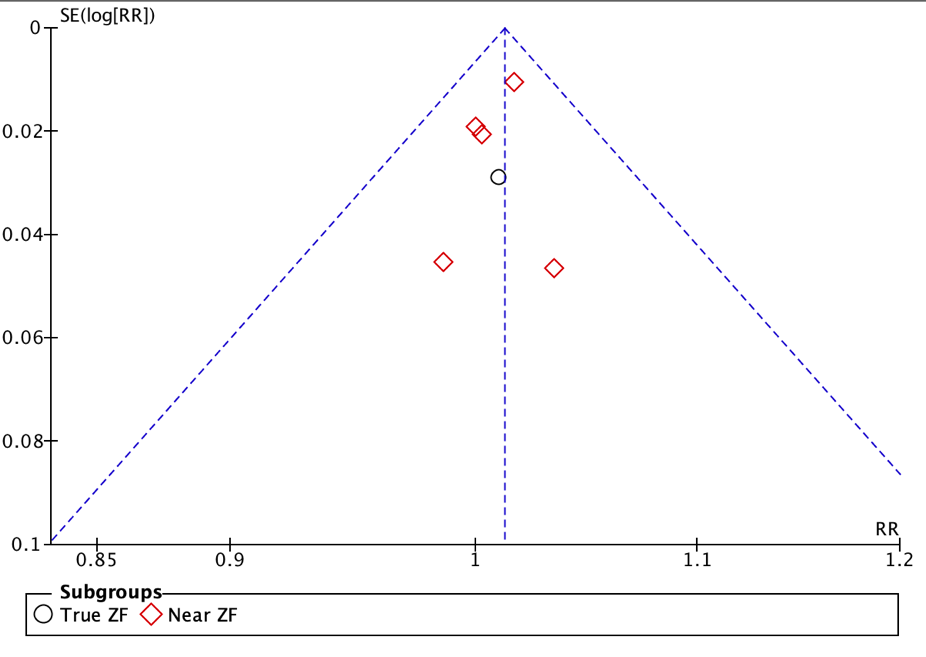


(C) Forrest plot of arrhythmia recurrence based on 3D-EAM application


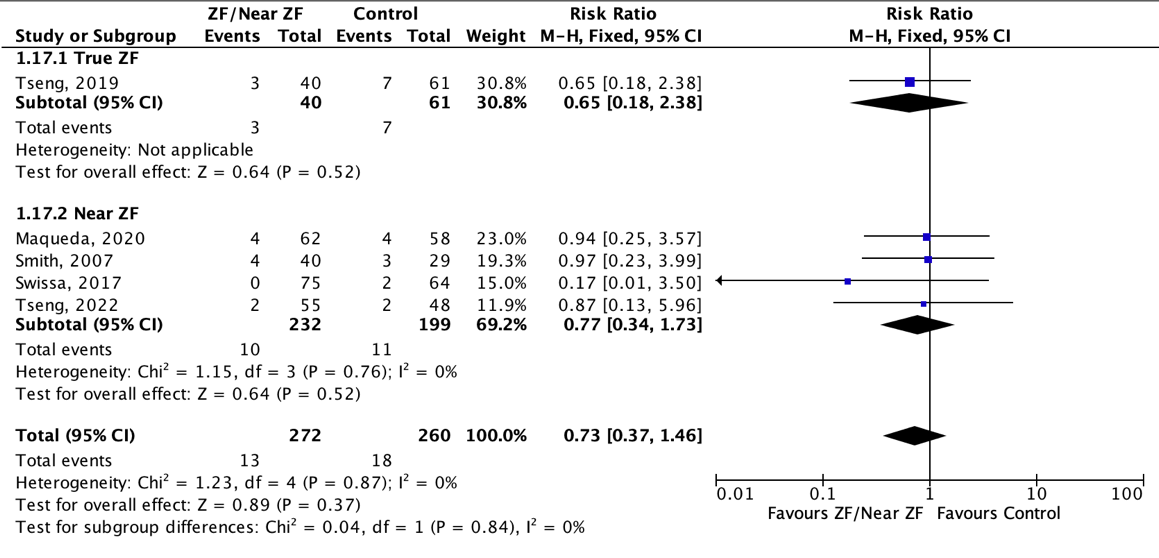


(D) Funnel plot of arrhythmia recurrence based on 3D-EAM application


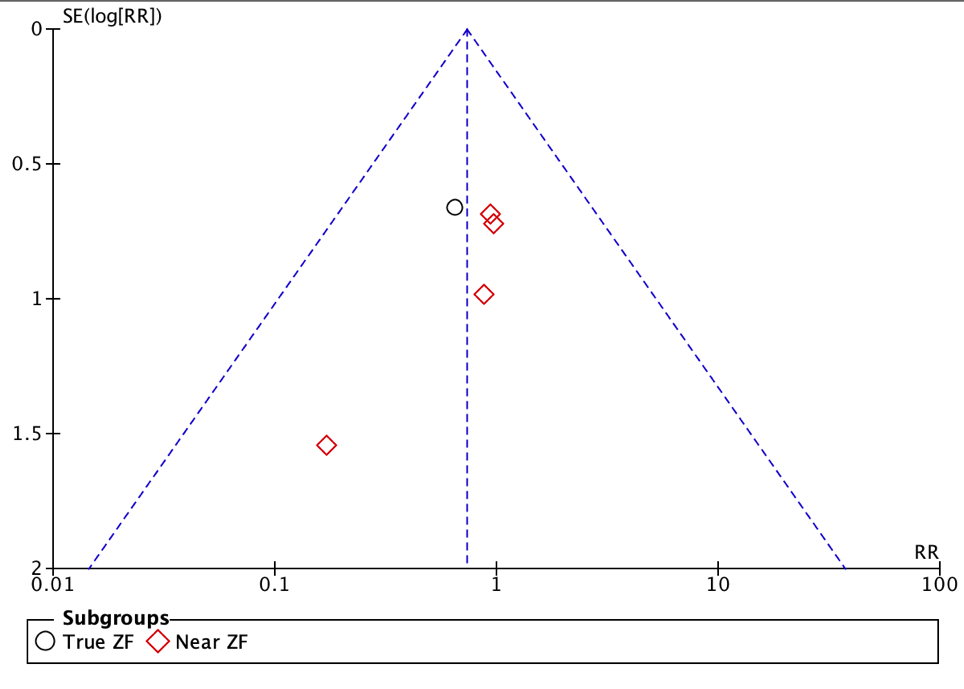


(E) Forrest plot of complication occurrence based on 3D-EAM application


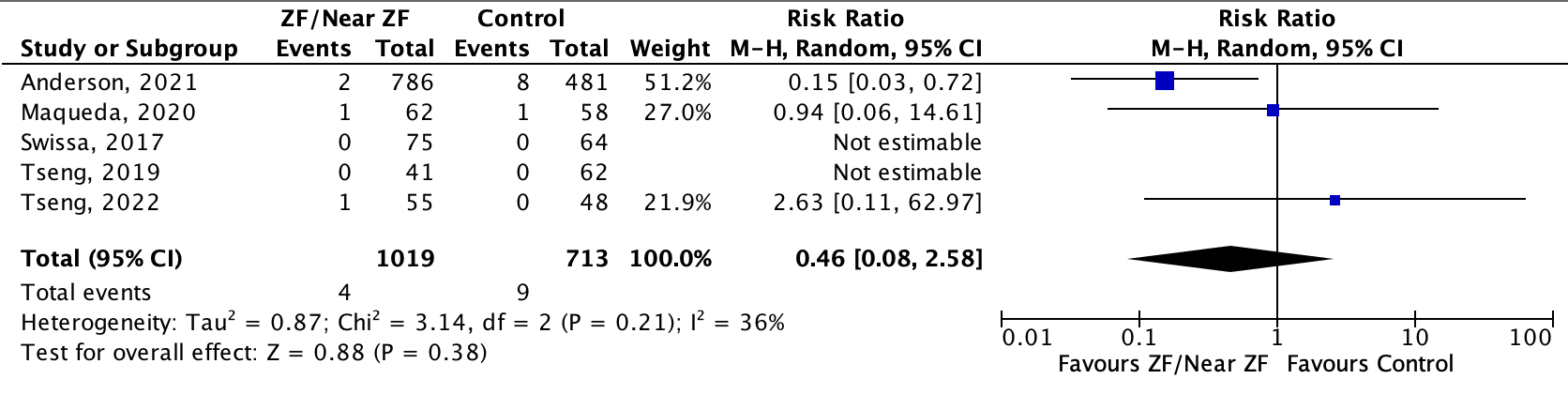


(F) Funnel plot of complication occurrence based on 3D-EAM application


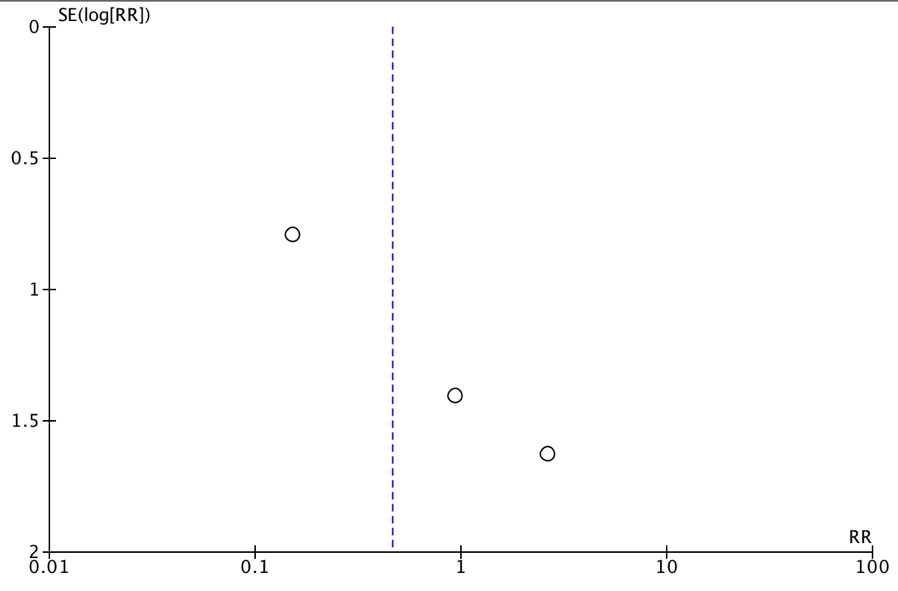

Supplement: Supplementary file 1 — Data S1: Supporting Information. [file JOA3-40-913-s001.docx]
